# Overdosed

**Published:** 2018-07-27

**Authors:** Tharshika Thangarasa, Usman Khan

**Affiliations:** 1 Faculty of Medicine, University of Ottawa, Ontario, Canada

**Figure:**
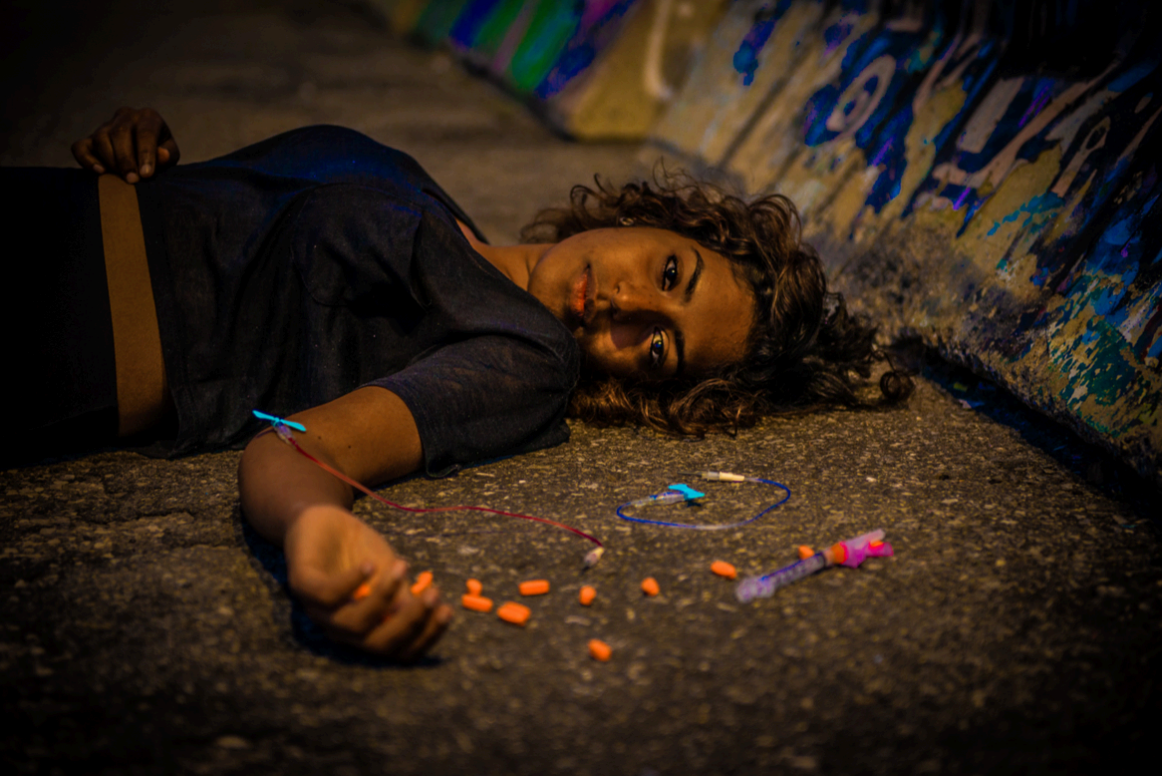


**Overdosed**.

You find me,
Sprawled across the cold, dark asphalt.
Incoherent, incompetent, “incapable”.
Hypotensive, bradycardic, cyanotic.

Overdosed.

For a moment, you pity me.
So young, likely to succumb to the effects
of the chemical concoction that I consciously
forced through my veins.

Overdosed.

Your pity subsides.
It makes way for disgust.
For judgement.
How could I have elected to end up this way?

Overdosed.

You walk around my limp, virtually lifeless body.
Assessing the patient,
As you have spent an eternity learning to do.
Through it all, my eyes stare right through you.

Overdosed.

Perhaps, fentanyl isn’t solely responsible for my state.
Perhaps, it was neglect, a faulty system.
But, judge as you may,
I overdosed.

Help.

The aim of this piece is to help highlight the stigma individuals with addictions often face within the medical system. We believe that it is fundamentally important that people consider the psychosocial factors that culminate in patients resorting to drugs and suicide as solutions. Before instinctually pointing fingers, and seeking to blame, one must at least try to appreciate the magnitude of the problem, and how it stretches beyond the individual. It is only then that we can even attempt to address the issues at hand.

## About the authors

Tharshika Thangarasa is a daughter, sister, friend, and third year medical student at the University of Ottawa. She loves playing with colours, art, and writing in her leisure time. She is passionate about mental health and thankful to be career that provides endless inspiration for her creative work.

Usman Khan is a third-year medical student at the University of Ottawa. Having served on the Board of Directors for the Canadian Federation of Medical Students, travelled almost fifty countries across the world, he hopes to pursue a career that involves leadership, global health, and surgery.

